# Organizing knowledge to enable personalization of medicine in cancer

**DOI:** 10.1186/s13059-014-0438-7

**Published:** 2014-08-27

**Authors:** Benjamin M Good, Benjamin J Ainscough, Josh F McMichael, Andrew I Su, Obi L Griffith

**Affiliations:** Department of Molecular and Experimental Medicine, The Scripps Research Institute, 10550 North Torrey Pines Road, La Jolla, CA 92037 USA; The Genome Institute, Washington University School of Medicine, 4444 Forest Park Ave, St Louis, MO 63108 USA; Department of Genetics, Washington University School of Medicine, 660 S. Euclid Ave, St Louis, MO 63110 USA; Department of Medicine, Washington University School of Medicine, 660 S. Euclid Ave, St Louis, MO 63110 USA

## Abstract

Interpretation of the clinical significance of genomic alterations remains the most severe bottleneck preventing the realization of personalized medicine in cancer. We propose a knowledge commons to facilitate collaborative contributions and open discussion of clinical decision-making based on genomic events in cancer.

## The bottleneck for realizing personalized medicine is now interpretation

The landscape of the genomics of tumor progression and heterogeneity has seen incredible advancements in recent years with the maturation of The Cancer Genome Atlas (TCGA) [1], International Cancer Genome Consortium (ICGC) [2] and other large-scale tumor sequencing efforts. Software and workflow systems for predicting and annotating genomic changes have proliferated and continue to improve [3]. Caregivers in the healthcare system will soon be faced with a large number of genomic alterations that are potentially relevant to understanding cancer progression and improving clinical decision making for each individual patient. However, there are few resources to help with the prioritization and interpretation of these alterations in a clinical context. Genomic events and the genes or pathways that they affect must be placed in the context of drug-gene or drug-variant interactions and associations with diagnostic or prognostic endpoints. The evidence for these associations must also be captured and characterized to allow risk-benefit analysis for any proposed clinical action. The bulk of this information remains trapped in the masses of published data, clinical trial records, and domain-specific databases. Sifting through this mountain of information is now the most critical bottleneck to making personalized medicine a reality in cancer. In this Opinion article, we propose the creation of a comprehensive, current, and community-based knowledge base to connect cancer genome events with the necessary evidence to evaluate their biological and clinical significance. Such a framework will allow the harnessing of collaborative contributions and open discussion needed to empower the most informed genomics-based clinical decision-making in a rapidly changing landscape.

Cancer genomics promises to revolutionize medicine by identifying tumor-specific alterations that can guide clinical decision-making. To list just two groundbreaking examples, activating mutations in the epidermal growth factor receptor gene *EGFR* were linked to gefitinib response [4,5] and amplification or overexpression of the related gene *ERBB2* was shown to predict response to anti-ERBB2 therapies such as lapatinib [6]. Tests for these markers that guide therapy decisions are now part of the standard of care in non-small-cell lung cancer and breast cancer. Since these and other early single-gene findings, large-scale sequencing studies have systematically mapped the landscape of the most common alterations for most common tumor types [1,2]. Increasingly, these alterations are being linked to diagnostic, prognostic, and drug-response outcomes. As the number of these associations increases and sequencing costs decrease, targeted panels are being replaced by genome- and transcriptome-wide approaches. Several proof-of-principle studies have recently demonstrated the potential for use of such data to identify clinically actionable findings [7–9]. In a prototypical study, Jones *et al.* [10] sequenced an oral adenocarcinoma by whole-genome and whole-transcriptome sequencing, identified upregulation of the mitogen activating protein kinase pathways through overexpression of receptor tyrosine kinase (RET) RNA and deletion of the Phosphatase and tensin homolog (*PTEN*) gene. They proposed a therapeutic intervention by RET inhibition with sunitinib, a therapy that might not otherwise be considered for this disease type. Most recently, Van Allen *et al.* [11] described an exome sequencing approach that, when applied prospectively, identified clinically relevant alterations in 15 of 16 cancer patients analyzed.

**Figure 1:**
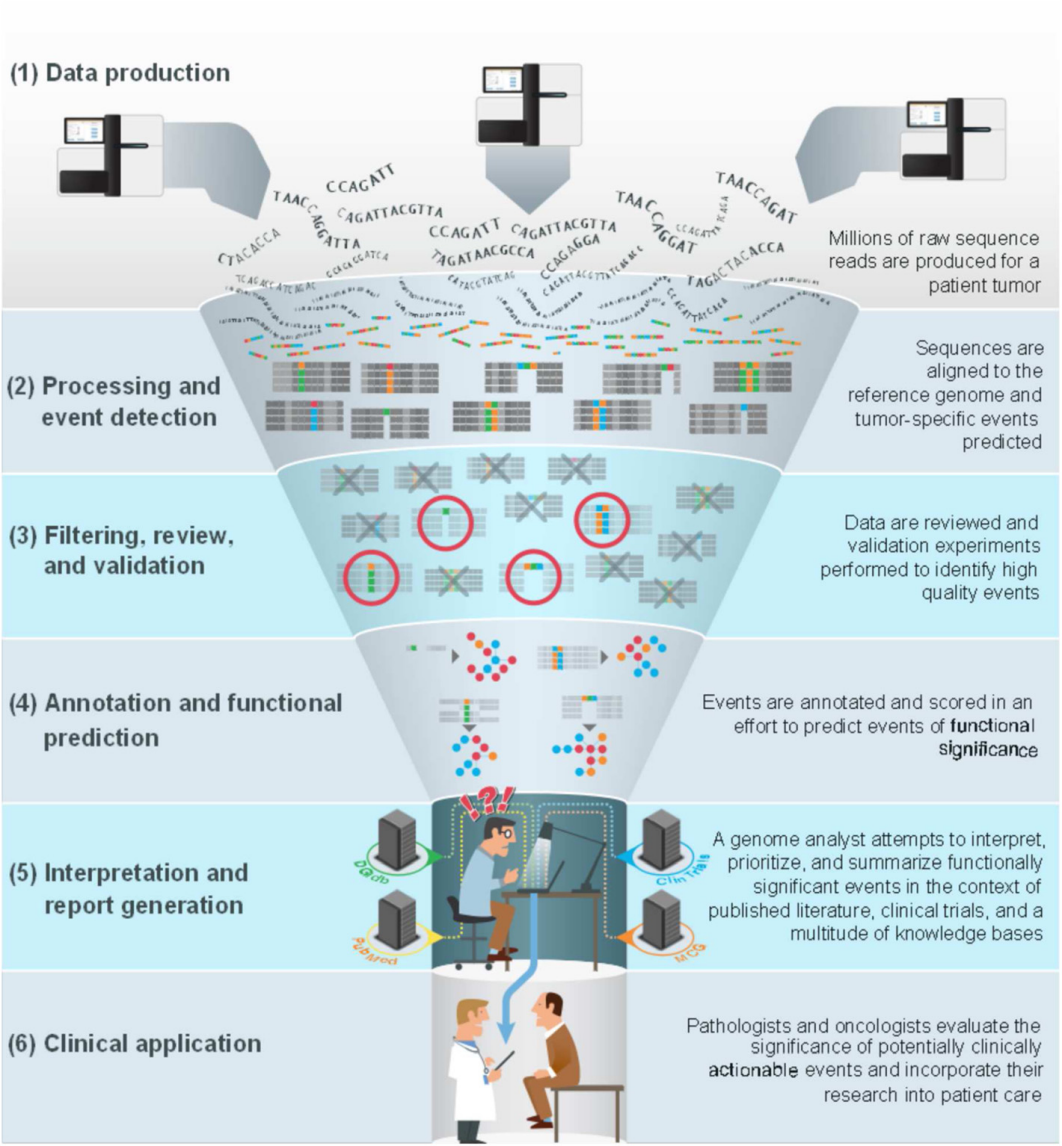
**The interpretation bottleneck of personalized medicine.** A typical cancer genomics workflow, from sequence to report, is illustrated. The upstream, relatively automated steps (shown by their light color here) involve (1) the production of millions of short sequence reads from a tumor sample; (2) alignment to the reference genome and application of event detection algorithms; (3) filtering, manual review and validation to identify high-quality events; and (4) annotation of events and application of functional prediction algorithms. These steps culminate in (5) the production of dozens to thousands of potential tumor-driving events that must be interpreted by a skilled analyst and synthesized in a report. Each event must be researched in the context of current literature (PubMed), drug-gene interaction databases (DGIdb), relevant clinical trials (ClinTrials) and known clinical actionability from sources such as My Cancer Genome (MCG). In our opinion, this attempt to infer clinical actionability represents the most severe bottleneck of the process. The analyst must find their way through the dark by extensive manual curation before handing off (6) a report for clinical evaluation and application by medical professionals.

These anecdotal examples hint at the promise of personalized (‘N-of-one’) medicine to target therapies to the specific genomic alterations of each cancer patient. A typical cancer genomics workflow is depicted in Figure 1. This process has been reviewed elsewhere extensively [11–13] and is arguably converging on some level of standardization and automation. The major bottleneck in the process currently lies in the final steps of interpretation and report generation. The challenge is to determine the significance of tumor-specific genomic changes in both a biological and clinical context. A large number of algorithms have been developed to predict the biological effects of single nucleotide variants (SNVs) and to a lesser degree insertions and deletions (indels). The overall accuracy of these methods is generally low [14] and very little has been done for other event types such as chimeric transcripts and copy number variants (CNVs).

Because computational predictions are inadequate, this challenge of biological and clinical interpretation of genomic events is primarily a challenge in knowledge management. There is a finite collection of knowledge about these events in the biomedical literature, and every cancer genome analyst desires access to the entirety of that knowledge in a concise and consumable form. When analysts reach the interpretation step in Figure 1, each potential tumor-driving event is typically evaluated manually against a disparate set of data sources. For example, candidate fusions might be evaluated against the Mitelman database of chromosomal alterations [15], Cancer Gene Census [16], the Gene Ontology [17], and drug-gene databases such as DGIdb [18]. Similarly, a subset of clinical associations for cancer have been catalogued in databases such as My Cancer Genome (MCG) [19], and variants associated with genetic diseases are recorded in resources such as ClinVar [20] and HGMD [21]. Although resources such as these are valuable, the fragmentation of this knowledge in uncoordinated and overlapping efforts is highly inefficient. And given that these efforts do not share a common set of standards and many are proprietary, the products of each group cannot be integrated easily.

We, as a community, need to create a collective resource for this knowledge. Information linking cancer genomic events to clinical interpretations and recommendations needs to be stored, retrieved, edited, and discussed. Only through such a shared knowledge structure will we realize the opportunities for personalized care raised by genomic technology. This knowledge base must be comprehensive. It must incorporate information now distributed across many different databases, scattered through the scientific literature, clinical trials reports, abstracts from conference proceedings, US Food and Drug Administration (FDA) reports and more. At the same time it must be focused. We need to capture detailed evidence for putative connections between genomic events in cancer and their interpretations. This evidence should be captured in structured forms and synthesized in detailed textual summaries that provide biological and clinical interpretations associated with particular genomic events. It must also be kept current. Our collective understanding of clinically important genomic events grows on a daily basis, and the knowledge base should aim to incorporate these advances as they arise.

How can we create and maintain a comprehensive, well-structured knowledge base that captures the relevant findings from hundreds of thousands of new sources each year, as well as the potentially varying interpretations of these findings by thousands of experts? We propose that the only plausible way to achieve this vision is for the community to become the primary contributors of content. Small professional teams can produce excellent resources but they cannot scale with the exponential growth in biomedical knowledge [22]. Of course, numerous attempts have been made in this direction - the great majority of which have failed to attract the critical mass of user contributors needed to thrive. However, there are successes that can be followed, and in the case of N-of-one cancer genomics we have a unique situation that is particularly well suited to a community model.

Every team tasked with an N-of-one analysis now already goes through the process of aggregating content from multiple databases, manually searching through large textual resources such as PubMed and ClinicalTrials.org, identifying relevant content, and translating that content into structured assertions. Dienstmann *et al.* [23] describe how their team gradually accumulates the information they use to form clinical interpretations of cancer genomes in what they term their ‘knowledge database’. In addition, they have taken the exemplary step of sharing that knowledge with the public via the Sage Synapse biology information commons [24] (in the form of a spreadsheet [25]). Many groups in both academia and industry are currently creating their own internal version of this knowledge base. Nearly all of this work is redundant. If we can convince the community to externalize even a few of the knowledge bases they are already assembling, as Dienstmann *et al.* [23] have done, we can as a community begin taking real steps in the direction of a common knowledge platform for cancer genomics. The first step is simply to make the choice that knowledge of this nature should remain free.

## Principles for building a successful community knowledge base

N-of-one teams need comprehensive access to data linking genes and specific genomic events to diagnostic, prognostic, and treatment information. Such links need to be annotated with detailed chains of evidence leading back to their original sources. Collated effectively, this information could greatly improve the pace with which high quality reports could be assembled. As much as possible, such annotation should aim for highly specific and unambiguous descriptions of events and use appropriate ontologies. For example, this might include the use of Human Genome Variation Society notation where possible for variants [26], the Disease Ontology [27] to specify cancer type and subtype, and the Sequence Ontology [28] or Variation Ontology [29] to describe event types. Tables 1, 2 and 3 summarize a proposed data model for the curation of evidence for clinical actionability of genomic events broken down into (1) evidence details, (2) event types, and (3) evidence types and levels.

**Table 1:** **A draft proposal for the minimal data needed for curation of evidence of a clinically actionable genomic event: evidence details**

**Data type**	**Description**	**Example**
Gene	Gene implicated (Entrez gene id)	*ESR1* (2099)
Event (gene-level or variant-level)	Genomic event such as SNV, indel, CNV, chimeric transcript, structural variation, epigenetic alteration, expression change, etc. See Table 2 for more details	chr6:g.152419922 T > A (Y537S)
Disease	Specific disease or disease subtype that is associated with this event and its clinical implication (Disease Ontology Identifier)	Estrogen-receptor positive breast cancer (DOID:0060075)
Evidence type	Category of clinical action implicated by event. See Table 3 for more details	Predictive
Evidence level	Levels of evidence for clinical actionability. See Table 3 for more details	Level B - clinical evidence
Evidence direction	A positive or negative value indicating whether the evidence statement supports or refutes a clinical association with the event	Positive - the evidence supports the association
Treatment (FDA status)	For predictive evidence, indicates the therapy for which sensitivity or resistance is indicated	Hormone therapy resistance
Actionability direction	Positive or negative association with treatment or diagnostic/prognostic end point	Negative - mutation is associated with resistance to therapy
Text summary (wiki-like)	Human readable interpretation. Free-form text summary of this event’s effect on cancer and potential clinical interpretations. This interpretation is the synthesis of all other information about an event and its relevance to clinical action and should be the living product of active discussion	Studies suggest ligand-binding-domain *ESR1* mutants mediate clinical resistance to hormonal therapy and suggest that more potent estrogen receptor antagonists may be of substantial therapeutic benefit
Source	Literature where the event is described/explored (PubMed id)	PMID: 24185512

Note: Example data were drawn from a single study describing evidence for the clinical relevance of ESR1 Y537S mutations.

**Table 2:** **A draft proposal for the minimal data needed for curation of evidence of a clinically actionable genomic event: types of events**

**Event type**	**Description**	**Example**
Single nucleotide variant (SNV)	Single nucleotide alterations	*BRAF* c.1799 T > A (V600E)
Small insertion or deletion (Indel)	Small numbers of nucleotides deleted or inserted	*PTEN* c.800delA (K267fs*9)
Copy number variation (CNV)	Large-scale (for example, chromosomal) or focal changes in copy-number status such as amplifications and deletions	*ERBB2* amplification
Structural variation (SV)	Large-scale (for example, chromosomal) rearrangements such as translocations or inversions	*FLT3* internal tandem duplication
Chimeric transcript	Aberrant expression of messenger RNA involving distant intra- or inter-chromosomal gene pairs	BCR-ABL fusion
Epigenetic modification	Alterations at the epigenetic level such as DNA methylation or histone modifications	*TERT* promoter hypermethylation
Expression biomarker	Significantly increased or decreased expression of RNA or protein	High SPARC expression

Note: Certain types of events are by their nature non-specific in the genomic sense. For example, there can be an almost infinite number of ways to truncate and thereby destroy function of a protein, such as the retinoblastoma protein. Many specific deletions in the *RB1* gene might be grouped together under a common generic event for ‘*RB1* loss’ with a consistent interpretation. Therefore, hierarchical relationships must be supported and ontologies may need to be modified or developed specifically for this domain space.

**Table 3:** **A draft proposal for the minimal data needed for curation of evidence of a clinically actionable genomic event: evidence types and levels**

**Evidence property**	**Evidence sub-property**	**Description**	**Example**
Type of evidence	Predictive	Genomic alteration is predictive of response to therapy	Breast cancer cell lines with *H1047R* mutation showed increased sensitivity to CH5132799 compared to cells with wild-type *PIK3CA* gene
	Diagnostic	Genomic alteration is diagnostic for disease or subtype	*DNAJB1:PRKACA* fusions are very strongly associated with the fibrolamellar variant of liver cancer
	Prognostic	Genomic alteration is prognostic for disease outcome	The presence of *KRAS* mutations in acute myelogenous leukemia is associated with shorter survival time
Level of evidence	A - validated association	Proven/consensus association in human medicine	A meta-analysis of clinical studies showed that harboring a *BRAF* V600E mutation predicts worse prognosis in patients with colorectal cancer
	B - clinical evidence	Clinical trial or other primary patient data supports association	In non-small-cell lung cancer patients with EGFR T790M and other activating mutations, their progression-free survival is shorter than those who do not have T790M mutations
	C - preclinical evidence	*In vivo* or *in vitro* models support association	Experiments showed that AG1296 is effective in triggering apoptosis in cells with the *FLT3* internal tandem repeat
	D - inferential association	Indirect evidence	Glioma cells harboring *IDH1* mutation may be more susceptible to chemotherapy or radiotherapy due to their reduced ability to respond to oxidative stress

Note: The schema for evidence types and levels was inspired by Van Allen *et al.* [11].

When considering community-generated knowledge bases that have succeeded, Wikipedia stands at the top of the list by far. Although there are undoubtedly a large number of reasons for its success, one distinguishing characteristic is its almost complete lack of gatekeepers. Anyone can edit a Wikipedia article. One powerful result of this openness is that many different types of people with different experience and inclinations can participate [30]. Some produce new text, some fix typos, some add images, some fix references, some write training material, some focus on single articles they care about, while others make minor improvements to thousands of articles. Systems with gatekeepers (for example, any database that says “please email the curators if you have something you would like to add”) make such a diversity of contributors unlikely.

The first principle that we suggest for the collective cancer genomic knowledge base is complete openness. Anyone should be able to add and edit content. This principle not only removes the inevitable slowdown caused by mandatory, top-down curatorial review of all changes, it also facilitates a diversity of ways that people with different kinds of skills can contribute. As an example, one team of clinical researchers might share a spreadsheet of claims linking genomic events to clinical interpretations. A bioinformatician might improve that contribution by exporting it as a comma-separated values (csv) file and replacing the gene and variant names with standard identifiers. Another person with understanding of the shared knowledge-based system might then be able to import that content. Yet another person might notice that there was an error in a particular interpretation and then make a change to the knowledge base. The key thing to note is that these roles can be decoupled across multiple people and even multiple teams. Rather than placing the entire burden on a single individual, this system facilitates iterative and sequential improvement of any contributed content.

Given a gatekeeper-less model, substantial attention must be paid to tracking the provenance of the claims that make their way into the knowledge base. Again, Wikipedia and its underlying MediaWiki software provide a model example. Every edit in Wikipedia is tracked, linked to a user or an IP address, and can be easily reverted. The edit history of an article and of an editor tell a story that can be used to reliably assign trust to either [31]. This information should be made accessible to applications that build on the knowledge base (for example, to generate reports) such that analysts can make their own decisions about whom to trust and for what reasons. In addition to allowing manual and computational decisions about trustworthiness of content, tracking contributions opens up the possibility of using earned reputation as a way of incentivizing contributions.

A final example from Wikipedia, already reflected in the model proposed in Table 1 and a requirement of a gatekeeper-less system, is the requirement for evidence. One of the tenets of the Wikipedia community is that every putatively factual statement should be supported by one or more external sources [32]. This is one of the key factors in making it the reliable resource that it generally is [33]. Readers can always look up the citations associated with a claim and make up their own mind. Any reader who disagrees with the stated claim can edit the article, provided that they too can offer external evidence. This evidence for claims is, of course, much more important in clinical situations. Once an analyst has used the knowledge base to hone in on a small set of events on which to base their interpretations, the next step is for them to examine the list of references produced (such as a list of PubMed identifiers and clinical trials records) and make their own judgment.

## Incentives for contributions to community resources in science

A common criticism of any proposal for a community-driven scientific resource is that scientists will not contribute. Reasons include firstly that there is no direct career incentive to do so, secondly that they are too busy already with work that does have career rewards, and thirdly that by sharing their work openly they could lose valuable competitive advantages. However, this issue of the need for career-based incentives to motivate scientific effort is not universal and, in fact, research has shown that it is largely false. Mazumder *et al.* [34] found that a lack of time (and not a lack of incentives) is the chief factor limiting researchers from contributing to open resources. This contention is further supported by the success of efforts to streamline the processes involved in contributing to open resources. As an example, Flybase increased their rate of community contributions sevenfold by introducing a proactive model in which database curators directly emailed authors of relevant papers [35], and a similar effort and result was observed with WormBase [36]. In addition to these community-based extensions to curated databases, there are multiple successful community knowledge platforms in the life sciences. Examples of these include the Encyclopedia of Life [37], the SEQanswers forum [38], the Gene Wiki [31,39], the integration of RFAM with Wikipedia [40], and the BioStar question and answer system for bioinformatics [41]. Each of these efforts has attracted and sustained large communities of active contributors. These results demonstrate that scientists will contribute to effectively designed community content curation efforts without any need for dramatic sociological shifts in the scientific incentive structure.

A community knowledgebase will also tap directly into career advancement incentives for cancer genome analysts. If implemented well, the proposed knowledge management system should allow analysts to add content directly to the centralized resource faster than they can assemble and maintain their own internal repositories. Integration of external databases could be accomplished by a few researchers rather than redundantly by everyone. Analysts could record clinically actionable genomic events through interfaces that specifically facilitate this kind of curation (for example, by supporting autocomplete fields that use shared identifiers and vocabularies). Analysts could in turn integrate this community content into their own genome interpretation pipelines. This system could be seeded with enough content to attract the attention of a cancer genomics community clearly starved for such resources. Ideally, this would be the start of a powerful positive feedback loop in which content was curated into the resource, thereby increasing the value of the resource and attracting more users, who in turn add more high quality content. Embedding community contribution seamlessly into the process of completing personal tasks directly addresses the issue of time constraints and directly incentivizes high quality contributions.

We also suggest that a system that is dedicated to remaining an open free public resource will attract a large amount of interest and contributors, particularly those outside of the traditional research enterprise. For example, as patients increasingly become better informed they have the desire, and in many cases also have the ability, to make contributions towards finding cures. This community may be particularly motivated in an area with as much direct clinical relevance as cancer genomics and thus could provide an extremely ‘long tail’ of curators for this initiative.

**Figure 2:**
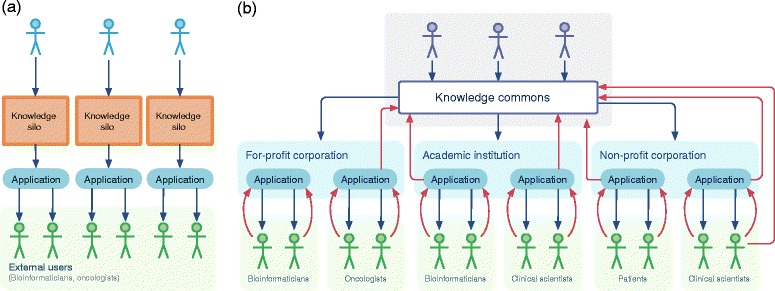
**An open, shared knowledge commons for N-of-one cancer researchers. (a)** The closed model of knowledge management. Nearly all corporations and even most academic and non-profit groups tend by default to set up closed systems in which users of the information have little incentive or mechanism to feed information back into a community resource. **(b)** The open knowledge model. A knowledge commons enables the development of a diverse community of applications targeted at different user groups. All users have the incentive to feed information back to the commons and apps can provide mechanisms to do so.

The proposed data model in Table 1 and the desired open participatory architecture described above are a rough requirements list for the knowledge base that we propose. The implementation of this system should enable the inclusion of both structured data and unstructured text, should track the provenance of all statements automatically, and should support read/write access by an application programming interface (API) as well as full data exports. Many of these features might be implemented through a combination of existing technologies and standards. Semantic MediaWiki provides one potential framework for collaborative management of both structured and unstructured knowledge [42]. Such a system should be directly integrated with standards for representing scientific claims and evidence [43], tracking information provenance [44], and for uniquely identifying core data elements such as genomic events [26] and disease types [27]. This technology stack should not only enable direct human interaction, but should also provide an effective API to stimulate a diverse array of applications that both consume the content and enable users to feed value back into the knowledge base directly (Figure 2).

## Complementary efforts: moving towards a universal ‘network of biothings’

Nascent large-scale initiatives, such as the global alliance for genomics and health [45] (GA4GH) and ClinGen [46], have been announced to help address a variety of information management problems related here. In particular, they emphasize the development of standards for structuring clinical genetic information for representation in electronic medical records and for deposition in the NCBI’s curated clinical genetic database ClinVar [20]. The visions behind these proposals represent substantial improvements over the current landscape of public information sources for medical genetics.

As these longer-term initiatives unfold, we expect that the bottom-up, community model proposed here will provide an immediately useful resource and will contribute to achieving the shared vision of effective knowledge management for personalized medicine. As GA4GH and ClinGen work to define standards and protocols in a top-down manner, the community can work from the bottom up to share information through the proposed knowledge base. The two initiatives should reinforce each other. The community knowledge base should accept and work towards implementing standards that will be decided on by the expert working groups of ClinGen and GA4GH while at the same time contributing evidence to their discussions and solving real, pressing problems in the interim.

Many other groups are already putting enormous effort into synthesizing the crucial knowledge needed to make effective clinical recommendations and, as Dienstmann *et al.* [23] exemplify, they are often willing to share this work with the rest of the community. Unfortunately, no existing system provides an effective way to capture and redistribute the ongoing efforts of these teams in a computationally useful way. We have proposed the creation of an open-access, open-source knowledge base to address the challenges of personalized medicine in cancer. This proposal arises, in part, from a recent initiative with the aim of assembling a more general ‘Network of Biothings’ (NoB) that spans many related problems in biology and medicine [47]. Here we have specifically focused on constructing a NoB for the N-of-one cancer challenge. This NoB should capture the evidence for clinically actionable genomic events as described in Tables 1, 2 and 3. To succeed, it must also meet certain criteria. First, it must be committed to remain an open resource. Numerous closed solutions are being developed in industry to tackle this problem. We need the open alternative. Second, it must stay current. This is a critical and perhaps the most serious challenge. New relevant data, reports, clinical trials and so on join the landscape every day and must be incorporated into the resource in a timely manner. Finally, it must be computable. The NoB should follow the principles of the Semantic Web [48] in terms of standard data formats, the application of ontologies and the distribution of data via public web APIs.

The N-of-one cancer genomics challenge stands as the tip of the spear in the march towards personalized medicine. Given the complexity of the disease(s), the gravity of the situation for patients, and the limited time available to make decisions, this challenge will continue to test the boundaries of what is possible. The tools created for this case, such as the community knowledge base proposed here, will stand as powerful examples for the many other clinical applications of genomic technology on the horizon.
